# Evaluation of nationwide variation in post-haemorrhagic ventricular dilatation management following national guideline implementation in the Netherlands

**DOI:** 10.1007/s00381-026-07477-5

**Published:** 2026-09-25

**Authors:** J. P. F. Bertens, P. A. Woerdeman, L. S. de Vries, G. E. van den Bosch, S. J. Steggerda, J. K. H. Spoor, L. S. Smit, D. R. Buis, R. W. Koot, M. W. Aalbers, H. R. Jeltema, J. van Aalst, N. E. van der Aa, M. L. Tataranno, L. C. Weeke, H. J. ter Horst, T. R. de Haan, R. J. Vermeulen, K. P. Dijkman, S. M. Mulder-de Tollenaer, E. Roze, O. H. J. Eelkman Rooda

**Affiliations:** 1 Division of Paediatric Neurosurgery, Department of Neurosurgery, Sophia Children’s Hospital, Erasmus University Medical Centre, Rotterdam, The Netherlands; 2 Department of Neurosurgery, Amsterdam University Medical Centre, Amsterdam, The Netherlands; 3 Division of Neuroscience, Department of Neurology and Neurosurgery, Paediatric Neurosurgery Wilhelmina Children’s Hospital, University Medical Centre Utrecht, Utrecht, The Netherlands; 4 Division of Neonatology, Department of Paediatrics, Willem-Alexander Children’s Hospital, Leiden University Medical Centre, Leiden, The Netherlands; 5 Division of Neonatology, Department of Neonatal and Paediatric Intensive Care, Sophia Children’s Hospital, Erasmus University Medical Centre, Rotterdam, The Netherlands; 6 Division of Paediatric Neurology, Department of Neurology, Sophia Children’s Hospital, Erasmus University Medical Centre, Rotterdam, The Netherlands; 7 Division of Paediatric Neurosurgery, Department of Neurosurgery, Emma Children’s Hospital, Amsterdam University Medical Centre, Amsterdam, The Netherlands; 8 Division of Paediatric Neurosurgery, Department of Neurosurgery, Willem-Alexander Children’s Hospital, Leiden University Medical Centre, Leiden, The Netherlands; 9 Division of Paediatric Neurosurgery, Department of Neurosurgery, Amalia Children’s Hospital, Radboud University Medical Centre, Nijmegen, The Netherlands; 10 Division of Paediatric Neurosurgery, Department of Neurosurgery, Beatrix Children’s Hospital, University Medical Centre Groningen, Groningen, The Netherlands; 11 Division of Paediatric Neurosurgery, Department of Neurosurgery, Mosakids Children’s Hospital, Maastricht University Medical Centre, Maastricht, The Netherlands; 12 Department of Neonatology, Wilhelmina Children’s Hospital, University Medical Centre Utrecht, Utrecht, The Netherlands; 13 Division of Neonatology, Department of Paediatrics, Amalia Children’s Hospital, Radboud University Medical Centre, Nijmegen, The Netherlands; 14 Department of Paediatrics, Beatrix Children’s Hospital, University Medical Centre Groningen, Groningen, The Netherlands; 15 Division of Neonatology, Department of Paediatrics, Emma Children’s Hospital, Amsterdam University Medical Centre, Amsterdam, The Netherlands; 16 Department of Paediatrics, Mosakids Children’s Hospital, Maastricht University Medical Centre, Maastricht, The Netherlands; 17 Division of Neonatology, Department of Paediatrics, Máxima Medical Centre, Veldhoven, The Netherlands; 18 Division of Neonatology, Department of Paediatrics, Isala Women’s and Children’s Hospital, Zwolle, The Netherlands

**Keywords:** Post-haemorrhagic ventricular dilatation, Preterm infants, Clinical practice variation, National survey, Cerebrospinal fluid diversion

## Abstract

**Purpose:**

Post-haemorrhagic ventricular dilatation (PHVD) is a severe complication of germinal matrix–intraventricular haemorrhage in preterm infants and is associated with significant morbidity and adverse neurodevelopmental outcomes. In 2023, national recommendations for PHVD management were revised, but implementation has not been evaluated. We aimed to assess diagnostic and therapeutic practices across Dutch neonatal intensive care units (NICUs), quantify inter-centre variation, and provide a basis for a national PHVD registry.

**Materials and methods:**

A nationwide, cross-sectional survey was disseminated among representatives from nine Dutch NICUs and seven affiliated paediatric neurosurgical departments. Per centre, one paediatrician and paediatric neurosurgeon reported institutional practices regarding monitoring, cerebrospinal fluid (CSF) drainage thresholds, temporising interventions, permanent CSF diversion criteria, follow-up, and research priorities. Practices were compared to the Dutch guideline.

**Results:**

All NICUs and neurosurgical centres responded. PHVD case volume varied (< 5 patients/year in 3/9 centres; > 20 in 1/9). Diagnostic thresholds were ventricular index > 97th percentile and anterior horn width > 6 mm in 7/9 centres, with higher or additional criteria in 2/9. Initial temporising management consisted of serial lumbar punctures and ventricular access device (VAD) placement. VAD tap duration before permanent diversion was typically 4–6 weeks (6/7). Ventriculoperitoneal shunting was the preferred permanent intervention in all centres, with weight thresholds of 2.0–2.5 kg.

**Conclusion:**

Following implementation of a national guideline, PHVD management in Dutch NICUs demonstrates high consistency in core diagnostics and treatment principles, showing that national standardisation of PHVD care is feasible. Residual variation is concentrated in areas where evidence remains limited, identifying priorities for future collaborative research.

**Supplementary Information:**

The online version contains supplementary material available at https://doi.org/10.1007/s00381-026-07477-5.

## Introduction

Germinal matrix–intraventricular haemorrhage (GMH-IVH) is one of the most common forms of brain injury in preterm infants, particularly those born before 30 weeks of gestation [1–4]. Severe haemorrhage (grade III IVH) and periventricular haemorrhagic infarction (PVHI, historically classified as grade IV IVH) represent the most severe manifestations of this condition. Both grade III IVH and PVHI are frequently complicated by post-haemorrhagic ventricular dilatation (PHVD), a major determinant of neurological outcome in this population [5–9]. PHVD develops in approximately 25–50% of infants with severe GMH-IVH and may contribute to secondary white matter injury through increased intracranial pressure (ICP), impaired cerebral perfusion, and inflammatory processes. Early identification using cranial ultrasonography (cUS) and timely intervention are therefore essential to limit secondary brain injury in this vulnerable population [10–14].

Therapeutic strategies aim to limit progression of ventricular dilatation and, when possible, avoid the need for permanent cerebrospinal fluid (CSF) diversion. Temporising interventions include repeated lumbar punctures (LPs) and the use of ventricular access devices (VADs), whereas ventriculoperitoneal shunting (VPS) is generally reserved for persistent or progressive PHVD, after the acute stage [15, 16]. However, the optimal timing of intervention remains a subject of ongoing debate. Increasing evidence supports early, ultrasound-guided intervention at lower thresholds, such as a ventricular index (VI) > 97th percentile and an anterior horn width (AHW) > 6 mm. Early intervention has been associated with smaller ventricular volumes, reduced white matter injury, and lower shunt dependency compared with intervention at higher thresholds [17–21]. Nevertheless, international consensus regarding optimal treatment thresholds and surgical timing is still lacking, and practice variation persists both between and within countries [22–24].

In the Netherlands, care for very preterm infants with severe GMH-IVH and PHVD is concentrated within nine neonatal intensive care units (NICUs). To promote national alignment in management, diagnostic and therapeutic recommendations were introduced in 2015 and revised in 2023 [25]. However, it remains unclear to what extent these recommendations have translated into harmonised clinical practice across centres. Understanding current practice variation is essential for evaluating guideline implementation and identifying opportunities for further harmonisation of clinical care. In addition, greater uniformity in diagnostic and therapeutic approaches could provide a unique foundation for a national PHVD registry, facilitating collaborative research and enabling future multicentre intervention studies in this vulnerable population.

This study aims to provide a nationwide overview of current diagnostic and therapeutic practices for PHVD in Dutch NICUs following the recent guideline revision. Using a multidisciplinary survey, we sought to describe institutional practices, quantify inter-centre variability in diagnostic and intervention strategies, and explore the feasibility of establishing a national PHVD registry to support future evidence-based research.

## Materials and methods

### Design

This nationwide cross-sectional survey was conducted within the Post-Haemorrhagic Hydrocephalus Outcomes and Evidence Network for Infants (PHOENIX), a multidisciplinary collaboration of paediatric neurosurgeons, neonatologists, and child neurologists involved in the care of preterm infants with PHVD across Dutch NICUs.

### Participants

All NICUs in the Netherlands provide care for preterm infants with IVH and PHVD and were invited to participate, including seven centres with an in-house paediatric neurosurgical department. Each centre was asked to designate one paediatrician (neonatologist or paediatric neurologist) and, where applicable, one paediatric neurosurgeon to complete the survey on behalf of the department. Participants were instructed to complete the survey on behalf of their institution, ensuring data reflected institutional practices rather than individual clinician preferences.

### Data collection and analysis

The survey content was informed by a comprehensive review of the literature, existing national protocols, and expert opinion from PHOENIX members. The questionnaires were developed collaboratively within PHOENIX and pilot-tested for clarity and clinical relevance prior to distribution. Two separate questionnaires were developed for neonatologists and child neurologists (28 items) and paediatric neurosurgeons (25 items), and implemented using the Castor electronic data capture platform (Online Resource 1 and 2) [26]. Survey domains included diagnostic monitoring, CSF drainage thresholds, temporising interventions, criteria for permanent CSF diversion, follow-up strategies, interest in participation in a national PHVD registry, and prioritisation of future research topics. Within these domains, both questionnaires addressed items pertaining to patient volume, LPs, CSF cultures, VAD puncture frequency and duration, the healthcare professionals performing VAD punctures, and imaging and follow-up practices. The surveys were electronically distributed on November 14, 2025, followed by one reminder 2 weeks later. All participants gave informed consent prior to study participation. Responses were collected between November 2025 and February 2026.

Descriptive analyses were performed to summarise survey responses and identify overall patterns and trends across centres, focusing on observed differences in clinical practice rather than formal statistical comparisons. When responses differed between paediatricians and paediatric neurosurgeons from the same centre, the response retained for analysis was selected based on the discipline primarily responsible for the clinical decision or practice addressed by the survey item. Midpoint values were assigned to predefined patient volume categories to estimate annual patient numbers. For categories without an upper bound (e.g. > 20), the lower threshold of the category was used for estimation. Two centres without an in-house paediatric neurosurgical department were excluded from analyses of survey items related to neurosurgical decision-making, to avoid overrepresentation of the policy of the consulting neurosurgical centre.

## Results

### Survey response and participating centres

Responses were received from all nine Dutch NICUs, represented by neonatologists (*N* = 7) and child neurologists (*N* = 2). Among the seven university medical centres (UMCs) with a paediatric neurosurgical department, neurosurgical representatives from all centres responded.

### PHVD case volume and diagnostic assessment

The annual number of PHVD patients, including patients without intervention as well as those receiving temporary or permanent treatment, treated varied per centre. Three NICUs reported managing ≤ 5 patients per year, one reported 6–10 patients, two reported 11–20 patients, and one reported > 20 patients annually. Two centres were unable to reliably estimate their annual PHVD patient volume. Based on midpoint estimates, approximately 68 PHVD patients are treated annually across seven reporting NICUs, corresponding to 9–10 PHVD patients per reporting NICU per year.

Diagnostic assessment of PHVD relied primarily on evaluation of ventricular size using cUS. Initial routine cUS monitoring in infants with grade III GMH-IVH and PVHI was performed every 1–2 days in most NICUs (*N* = 6/9) and every 3–4 days in 3/9 centres. Radiological criteria constituted the primary basis for PHVD diagnosis in 9/9 centres, in accordance with the Dutch guideline, while one centre additionally incorporated clinical indicators, such as rapidly increasing HC or signs of raised ICP. Regarding ventricular measurements, 6/9 centres combined subjective assessment of ventricular enlargement with objective measurements (VI and AHW), whereas 3/9 centres based the diagnosis exclusively on objective measurements, reflecting guideline recommendations. Ultrasonographic thresholds used to define PHVD varied across centres: seven NICUs applied VI > 97th percentile and AHW > 6 mm, one centre additionally included thalamo-occipital distance (TOD) > 25 mm, and one centre used higher thresholds, defined as VI approaching the 97th percentile + 4 mm in combination with AHW > 10 mm.

### Criteria and timing for initiation of treatment

Across the seven centres with paediatric neurosurgical departments, the estimated annual number of patients undergoing a first CSF–diverting intervention ranged from ≤ 5 patients/year in 4 centres, 6–10 patients/year in 2 centres, and 11–20 patients/year in 1 centre, corresponding to an estimated total of approximately 42 patients annually. Treatment initiation was predominantly guided by objective ventricular measurements (VI and AHW) in six centres, consistent with the national guideline, whereas three centres additionally incorporated subjective assessment of ventricular enlargement. cUS to inform treatment initiation was performed every 1–2 days in eight centres, as recommended by the Dutch guideline, and every 3–4 days in one centre. The ventricular parameter thresholds employed to determine the timing of treatment initiation varied across centres and are shown in Table 1. Most thresholds corresponded to the Dutch guideline for early intervention, while a minority of centres applied higher, though still early, thresholds. The therapeutic objectives underpinning PHVD interventions are outlined in Table 2. These were largely guideline-concordant, except in three centres which additionally reported prevention of further ventricular dilatation and reduction of VI below p97 + 4 mm as intervention objectives.

**Table 1:** Criteria for PHVD therapy initiation

	*N*
VI > p97^a^	1/9
VI > p97 approaching + 4 mm	1/9
VI > p97 and AHW > 6 mm	3/9
VI > p97 approaching + 4 mm and AHW > 6 mm	3/9
VI > p97 approaching + 4 mm, AHW > 6 mm and/or TOD > 25 mm	1/9

*AHW *anterior horn width, *PHVD* post-haemorrhagic ventricular dilatation, *p97* 97th percentile, *TOD* thalamo-occipital distance, *VI* ventricular index

^a^[14]

**Table 2:** Objectives of interventions for PHVD

	*N*
Relief of clinical symptoms of increased ICP and/or attenuation of HC growth to < 2 cm/week, if present	2
Prevention of further ventricular dilatation	2
Reduction of ventricular size	3
Reduction of ventricular size, specifically VI < p97 + 4 mm	1
Reduction of ventricular size, specifically VI < p97	3
Reduction of ventricular size, specifically AHW < 6 mm	4

*AHW* anterior horn width, *HC* head circumference, *ICP* increased intracranial pressure, *p97* 97th percentile, *TOD* thalamo-occipital distance, *VI* ventricular index

### Temporising treatment strategies

Initial temporary management included serial LPs and VAD placement in 6/7 neurosurgical centres. One centre additionally reported neuro-endoscopic lavage (NEL) [27]. LPs were performed either supine or in a sitting position in seven centres and exclusively supine in two. The targeted volume of CSF per LP was 5–10 mL/kg in all centres, with three (*N* = 3/9), four (*N* = 1/9), and five (*N* = 4/9) LPs performed before considering alternative interventions. Notably, one centre reported up to six LPs. In all neurosurgical centres, VAD placement was the preferred temporary intervention prior to permanent shunting when serial LPs failed to stabilise PHVD. Collectively, these temporising practices closely reflect the stepwise approach recommended in the national guideline.

### VAD placement and management

Neurosurgical centres reported conditions for VAD placement to include performance of magnetic resonance imaging (MRI) and adequate patient weight, the latter not being stipulated by the Dutch guideline. VAD punctures were performed primarily by neonatologists (*N* = 8/9), residents (*N* = 6/9), and physician assistants or advanced nurse practitioners (*N* = 9/9) and less frequently by child neurologists (*N* = 3/9) and nurses (*N* = 3/9). No guideline recommendation exists regarding the healthcare professionals responsible for performing VAD punctures.

The frequency of VAD punctures was typically once (*N* = 1/7) or twice (*N* = 6/7) daily, concordant with national guideline recommendations. Following VAD punctures, CSF cultures were obtained two (*N* = 4/7) to three (*N* = 2/7) times per week in most centres, as advised in the national guideline, and once weekly in one centre. Ultrasonography to guide the extent of CSF drainage was performed daily (*N* = 3/7), every 2 days (*N* = 3/7), or twice weekly (*N* = 1/7). The guideline stipulates daily cUS during the first week, with frequency subsequently adjusted according to ventricular measurements. MRI was generally used to plan both temporary and permanent CSF diversion procedures (*N* = 3/7), while some centres reserved MRI for permanent CSF diversion procedures only (*N* = 2/7), or do not use MRI for either indication (*N* = 2/7). The Dutch guideline recommends considering MRI when ultrasound findings are suggestive of aqueduct stenosis or a trapped fourth ventricle, as these findings may preclude successful management by LPs alone.

The maximum duration of VAD punctures before transitioning to permanent CSF-diverting procedures was 4 to 6 weeks in most centres (*N* = 6/7) and 6 weeks in the remaining centre. Duration was contingent on gestational age (GA), adequate patient weight, and laboratory parameters, including CSF erythrocyte count and CSF protein concentration, being within normal range, in accordance with the national guideline. All centres performed a temporary challenge through discontinuation of VAD punctures for several days, prior to permanent CSF diversion, as recommended by the guideline.

### Permanent CSF diversion strategies

All seven neurosurgical departments reported VPS as the preferred permanent intervention. Criteria for transitioning from a VAD to permanent CSF diversion included patient weight (*N* = 6/7), CSF erythrocyte count (*N* = 4/7), and CSF protein concentration (*N* = 2/7), concordant with national guideline recommendations. Weight thresholds for VPS ranged from > 2 kg (*N* = 3/7) to > 2.5 kg (*N* = 4/7), reflecting guideline-recommended practice. Endoscopic third ventriculostomy (ETV) was considered a secondary treatment option in four centres for specific indications, including an obstructive component identified on MRI, while ETV was performed rarely or not at all in the remaining three centres [28]. Weight thresholds for ETV varied across centres, with most centres reporting a minimum weight of > 2.5 kg (*N* = 3/7). Three centres considered treatment at > 2, > 3.0, and > 3.5 kg, and one centre reported the weight threshold as unknown. The national guideline acknowledges ETV as a potential alternative to VPS in selected patients, while noting that there is currently no established role for ETV in neonatal PHVD management and that greater efficacy has been reported beyond 6 months of age. Table 3 summarises reported temporising CSF diversion, VAD placement and management, and permanent CSF diversion strategies across participating centres, alongside the corresponding Dutch national recommendations.

**Table 3:** National recommendations and reported practices for temporising treatment and permanent CSF diversion in PHVD

Management domain	Survey item	National recommendation	Reported centre practice, *n*/*N*
Temporising treatment strategies	CSF drainage by LPs	Maximum 10 mL/kg/day	Target CSF volume 5–10 mL/kg: 9/9
	Escalation following LPs	Consider ventricular reservoir placement after 4–5 LPs, or earlier if LPs are unsuccessful or poorly tolerated	Alternative intervention considered after 3 LPs: 3/9; 4 LPs: 1/9; 5 LPs: 4/9; 6 LPs: 1/9
	Temporary CSF diversion	VAD placement after unsuccessful LPs	VAD preferred temporary intervention: 7/7. NEL considered as alternative treatment option: 1/7
VAD placement and management	Initial VAD-tapping regimen	Usually 1–2 taps/24 h; initially 2 taps of 5 mL/kg, maximum 10 mL/kg per tap	Once daily: 1/7; twice daily: 6/7
	cUS monitoring	Daily cUS during first week after VAD placement, frequency subsequently adjusted according to ventricular measurements and reduced to approximately three times per week in stable infants	Daily: 3/7; every 2 days: 3/7; 2/week: 1/7
	CSF culture frequency	2–3 times/week	Once per week: 1/7; 2 times/week: 4/7; 3 times/week: 2/7
	Pre-procedural MRI	Consider MRI, or direct VAD placement, when cUS suggests aqueduct stenosis or trapped fourth ventricle	Prior to temporary and permanent procedures: 3/7; prior to permanent procedures only: 2/7; neither: 2/7
	Timing of transition to permanent CSF diversion	Reassess ongoing VAD drainage requirement after approximately 4–6 weeks. Consider GA, body weight, CSF erythrocyte count, and CSF protein concentration before starting permanent CSF diversion	Maximum VAD drainage duration 4–6 weeks: 6/7; maximum of 6 weeks: 1/7
Permanent CSF diversion strategies	Drain challenge	Temporarily stop VAD drainage before considering VPS placement	Performed in 7/7 centres
	Preferred permanent CSF diversion procedure	VPS	VPS: 7/7 centres
	Criteria for transition to permanent CSF diversion^a^	Ongoing need for CSF drainage; preferably consider CSF protein concentration, erythrocyte count and body weight	Body weight: 6/7; CSF erythrocyte count: 4/7; CSF protein concentration: 2/7
	VPS weight threshold	2.0–2.5 kg	> 2.0 kg: 3/7; > 2.5 kg: 4/7
	ETV	Alternative treatment option to VPS, effectiveness in the neonatal period may be limited, no current role in neonatal PHVD management. Possible effectiveness after 6 months of age	Alternative treatment option in selective cases: 4/7. Rarely or not performed: 3/7

*CSF* cerebrospinal fluid, *cUS* cranial ultrasonography, *ETV* endoscopic third ventriculostomy, *LP* lumbar puncture, *MRI* magnetic resonance imaging, *PHVD* post-haemorrhagic ventricular dilatation, *VAD* ventricular access device, *VPS* ventriculoperitoneal shunt

^a^More than one criterion could be reported by each centre

### Follow-up and outcome monitoring

Eight NICUs reported systematic long-term neurological and developmental follow-up for PHVD patients. Follow-up was primarily conducted through the neonatology department in six centres, and in two centres jointly through the neonatology and paediatric neurology departments. In the remaining centre, follow-up was limited to selected patients. Standardised assessments of cognitive and motor development included the General Movement Assessment (GMA) (*N* = 1/9), Movement Assessment Battery for Children (M-ABC) (*N* = 8/9), Bayley Scales of Infant and Toddler Development (BSID) (*N* = 9/9), the Wechsler Preschool and Primary Scale of Intelligence (WPPSI) (*N* = 8/9), and the Wechsler Intelligence Scale for Children (WISC) (*N* = 1/9), in accordance with the national neonatal follow-up guideline. In addition, systematic documentation of complications, including infections, haemorrhages, and shunt dysfunction, was performed in six of the seven neurosurgical centres.

### Perspectives on national PHVD registry and research priorities

All paediatricians and paediatric neurosurgeons from the nine participating NICUs, including seven centres with a paediatric neurosurgical department, expressed interest in participating in the establishment of a national PHVD registry to facilitate further healthcare quality improvement and research. Across both professional groups, the optimal timing of permanent CSF diversion was identified as a key priority for future PHVD research, while the prioritisation of other research topics differed between groups (Table 4). Additional proposed areas of research included lavage systems (e.g. IRRAflow), minimum thresholds for VAD placement in relation to birth weight and gestational age, NEL prior to VAD placement, pharmacological inhibition of CSF production, long-term follow-up after permanent CSF diversion, pressure-guided CSF drainage during LPs and VAD punctures, and the timing of drain challenge initiation [29].

**Table 4:** Survey-based priorities for future research in PHVD

	*N* (%)
**Paediatric neurosurgeons**	**7/16 (44%)**
1. Optimal timing of permanent CSF diversion	5/7 (71%)
2. Role of ETV in neonates	4/7 (57%)
3. Optimal timing of first intervention	3/7 (43%)
4. Inter-centre benchmarking (e.g. VPS rate, VPS revision rate, infections, short- and long-term neurological outcomes)	3/7 (43%)
5. Use of NEL	2/7 (29%)
6. Prediction of neurodevelopmental outcomes	2/7 (29%)
**Paediatricians (neonatologists and child neurologists)**	**9/16 (56%)**
1. Inter-centre benchmarking (e.g. VPS rate, VPS revision rate, infections, short- and long-term neurological outcomes)	8/9 (89%)
2. Optimal timing of permanent CSF diversion	6/9 (67%)
3. Prediction of neurodevelopmental outcomes	5/9 (56%)
4. Role of ETV in preterm neonates	3/9 (33%)
5. Optimal timing of first intervention	2/9 (22%)
6. Use of NEL	2/9 (22%)

*CSF* cerebrospinal fluid, *ETV* endoscopic third ventriculostomy, *NEL* neuro-endoscopic lavage, *PHVD* post-haemorrhagic ventricular dilatation, *VPS* ventriculoperitoneal shunt

## Discussion

This nationwide survey provides a comprehensive overview of current PHVD management practices across all Dutch NICUs. The findings indicate that PHVD management across Dutch NICUs is guided by early, imaging-driven monitoring and clinical decision-making in accordance with the Dutch guideline. At the same time, inter-centre variability persists, predominantly in domains where high-quality evidence remains limited. These findings are particularly relevant in the context of persistent international variation in PHVD management. Recent North American data demonstrate considerable heterogeneity in treatment pathways, including thresholds for intervention and approaches to CSF diversion [24]. In contrast, the present study shows that a nationally agreed, measurement-guided management framework can translate into highly concordant practice across an entire healthcare system. The Dutch experience therefore provides a real-world example of the feasibility of national standardisation in PHVD care, while the residual variation observed in areas not firmly addressed by evidence highlights where further comparative research is needed.

One of the most salient overarching trends is the reliance on serial cUS to guide both diagnosis and treatment initiation. The consistent use of standardised ventricular measurements reflects a broad national consensus that clinical manifestations, such as signs of raised ICP and increase in head circumference (HC), represent late-stage disease and are therefore unsuitable as primary triggers for intervention. Despite this shared diagnostic framework, small variations in imaging frequency and intervention thresholds suggest that the operationalization of early-intervention principles differs between centres, although reported values are mostly near recommended treatment thresholds [30]. Such findings underscore that remaining uncertainty primarily concerns the precise timing and criteria for intervention, rather than the value of early intervention itself.

Overall, several aspects of PHVD management were highly consistent across centres. Most centres reported a stepwise management strategy in which temporising measures, including serial LPs and VADs, are used prior to consideration of permanent CSF diversion. This uniformity suggests strong alignment in underlying treatment principles and is consistent with current recommendations aimed at stabilising ventricular dynamics, allowing time for recovery of physiological CSF circulation, and, in cases where this is not achieved, bridging to VPS placement once infants reach appropriate clinical conditions. In contrast, variability in CSF diversion protocols and criteria for escalation likely reflects the absence of high-quality comparative data, rather than divergent clinical intent.

Similarly, differences in criteria guiding the transition to permanent CSF diversion reflect the lack of neurosurgical consensus acknowledged in national recommendations. Although current guidelines do not assign a role to ETV in the management of PHVD in neonates, its application in selected centres suggests ongoing clinical uncertainty regarding patient selection and expected outcomes. In these centres, ETV is considered in carefully selected patients, rather than as a routine alternative to VPS [28]. This observation does not indicate endorsement of ETV efficacy in this population but rather highlights the need for clearer evidence to delineate its potential role, if any, beyond infancy.

Finally, the widespread provision of structured neurodevelopmental follow-up and the strong nationwide support for establishing a PHVD registry indicate broad recognition that further advances in care depend on coordinated national collaboration. Harmonised data collection, shared outcome evaluation, and sufficiently powered multicentre analyses are essential to address persistent uncertainties, particularly regarding the optimal timing of permanent CSF diversion, and to inform future guideline revisions. Moreover, harmonised registry-based data collection would facilitate more robust comparisons of PHVD incidence and outcomes across healthcare systems by ensuring consistent diagnosis and reporting of PHVD, with adjustment for differences in the underlying preterm population. In addition, such a registry may provide a unique platform for future multicentre intervention studies in this vulnerable population.

This study has several strengths, including its nationwide design with complete participation of all Dutch NICUs and its multidisciplinary perspective. However, some limitations should be acknowledged. Survey responses reflect reported institutional practices and may not fully capture variation at the individual clinician level. In addition, the relatively small number of centres, inherent to the Dutch setting, limits the ability to perform formal comparative analyses.

In summary, this nationwide study demonstrates that a nationally agreed, measurement-guided PHVD management strategy can be implemented with substantial consistency across an entire healthcare system. Residual variation is concentrated in areas where evidence remains evolving, identifying clear priorities for future collaborative, outcome-driven research.

## Supplementary Information

Below is the link to the electronic supplementary material.

ESM 1 (DOCX 35.5 KB)

ESM 2 (DOCX 36.4 KB)

## Data Availability

The authors are in full control of the primary data. Data generated and/or analysed during the study are available from the corresponding author upon reasonable request and can be reviewed by the journal if required.

## Abbreviations

AHW: Anterior horn width
BSID: Bayley Scales of Infant and Toddler Development
CSF: Cerebrospinal fluid
cUS: Cranial ultrasonography
ETV: Endoscopic third ventriculostomy
GMA: General Movement Assessment
GMH-IVH: Germinal matrix–intraventricular haemorrhage
HC: Head circumference
ICP: Intracranial pressure
LPL: umbar puncture
M-ABC: Movement Assessment Battery for Children
MRI: Magnetic resonance imaging
NEL: Neuro-endoscopic lavage
NICU: Neonatal intensive care unit
PHVD: Post-haemorrhagic ventricular dilatation
PVHI: Periventricular haemorrhagic infarction
TOD: Thalamo-occipital-distance
UMC: University medical centre
VAD: Ventricular access device
VI: Ventricular index
VPS: Ventriculoperitoneal shunt
WISC: Wechsler Intelligence Scale for Children
WMO: Medical Research Involving Human Subjects Act
WPPSI: Wechsler Preschool and Primary Scale of Intelligence
