# Supplementary material for: Evaluation of nationwide variation in post-haemorrhagic ventricular dilatation management following national guideline implementation in the Netherlands
Source: Childs Nerv Syst. 2026 Sep 25;42(1):375. doi: 10.1007/s00381-026-07477-5 (PMC13615105; doi:10.1007/s00381-026-07477-5)
Supplement: Supplementary file 1 [file 381_2026_7477_MOESM1_ESM.docx]

**ONLINE RESOURCE 1. SURVEY NEONATOLOGISTS AND CHILD NEUROLOGISTS**

**JOURNAL**

Child’s Nervous System

**TITLE**

Evaluation of Nationwide Variation in Post-Haemorrhagic Ventricular Dilatation Management Following National Guideline Implementation in the Netherlands

**AUTHORS**

J.P.F. Bertens^1,2^, P.A. Woerdeman^3^, L.S. de Vries^4^, G.E. van den Bosch^5^, S.J. Steggerda^4^, J.K.H. Spoor^1^, L.S. Smit^5,6^, D.R. Buis^7^, R.W. Koot^8^, M.W. Aalbers^9^, H.R. Jeltema^10^, J. van Aalst^11^, N.E. van der Aa^12^, M.L. Tataranno^12^, L.C. Weeke^12,13^, H.J. ter Horst^14^, T.R. de Haan^15^, R.J. Vermeulen^16^, K.P. Dijkman^17^, S.M. Mulder-de Tollenaer^18^, E. Roze^5*^, O.H.J. Eelkman Rooda^1*^.

**These authors contributed equally.*

**AFFILIATIONS**

^1^Division of Paediatric Neurosurgery, Department of Neurosurgery, Sophia Children's Hospital, Erasmus University Medical Centre, Rotterdam, The Netherlands

^2^Department of Neurosurgery, Amsterdam University Medical Centre, Amsterdam, The Netherlands

^3^Division of Neuroscience, Department of Neurology and Neurosurgery, Paediatric Neurosurgery Wilhelmina Children's Hospital, University Medical Centre Utrecht, Utrecht, The Netherlands

^4^Division of Neonatology, Department of Paediatrics, Willem-Alexander Children's Hospital, Leiden University Medical Centre, Leiden, The Netherlands

^5^Division of Neonatology, Department of Neonatal and Paediatric Intensive Care, Sophia Children's Hospital, Erasmus University Medical Centre, Rotterdam, The Netherlands

^6^Division of Paediatric Neurology, Department of Neurology, Sophia Children's Hospital, Erasmus University Medical Centre, Rotterdam, The Netherlands

^7^Division of Paediatric Neurosurgery, Department of Neurosurgery, Emma Children's Hospital, Amsterdam University Medical Centre, Amsterdam, The Netherlands

^8^Division of Paediatric Neurosurgery, Department of Neurosurgery, Willem-Alexander Children's Hospital, Leiden University Medical Centre, Leiden, The Netherlands

^9^Division of Paediatric Neurosurgery, Department of Neurosurgery, Amalia Children's Hospital, Radboud University Medical Centre, Nijmegen, The Netherlands

^10^Division of Paediatric Neurosurgery, Department of Neurosurgery, Beatrix Children's Hospital, University Medical Centre Groningen, Groningen, The Netherlands

^11^Division of Paediatric Neurosurgery, Department of Neurosurgery, Mosakids Children's Hospital, Maastricht University Medical Centre, Maastricht, The Netherlands

^12^Department of Neonatology, Wilhelmina Children’s Hospital, University Medical Centre Utrecht, Utrecht, The Netherlands

^13^Division of Neonatology, Department of Paediatrics, Amalia Children's Hospital, Radboud University Medical Centre, Nijmegen, The Netherlands

^14^Department of Paediatrics, Beatrix Children's Hospital, University Medical Centre Groningen, Groningen, The Netherlands

^15^Division of Neonatology, Department of Paediatrics, Emma Children's Hospital, Amsterdam University Medical Centre, Amsterdam, The Netherlands

^16^Department of Paediatrics, Mosakids Children's Hospital, Maastricht University Medical Centre, Maastricht, The Netherlands

^17^Division of Neonatology, Department of Paediatrics, Máxima Medical Centre, Veldhoven, The Netherlands

^18^Division of Neonatology, Department of Paediatrics, Isala Women's and Children's Hospital, Zwolle, The Netherlands

**CORRESPONDING AUTHOR**

JPF Bertens^1,2^, [J.p.f.bertens@amsterdamumc.nl](mailto:J.p.f.bertens@amsterdamumc.nl)

**ENGLISH TRANSLATION OF THE ORIGINAL DUTCH SURVEY**

Dear colleague,

We kindly invite you to complete this survey (on behalf of your centre*) with the aim of gaining insight into the current treatment practices for post-haemorrhagic ventricular dilatation (PHVD) in neonates in Dutch (university) medical centres. Your input will help to map variation in practice, identify potential bottlenecks in PHVD care, and facilitate the development of joint research initiatives and future guidelines. The survey consists of 28 questions and takes approximately 10–15 minutes to complete. You will also be given the opportunity to indicate whether you would like to be involved in future research initiatives, if you wish.

**We request each centre to designate one paediatric neurosurgeon and one neonatologist/paediatric neurologist to complete the survey on behalf of the department.*

Kind regards,

*Dr. O.H.J. Eelkman Rooda, paediatric neurosurgeon (Erasmus University Medical Centre and Princess Máxima Centre)
Dr. E. Roze, paediatrician–neonatologist and paediatric neurologist (Sophia Children’s Hospital, Erasmus University Medical Centre)
Dr. S.J. Steggerda, paediatrician–neonatologist (Willem-Alexander Children’s Hospital, Leiden University Medical Centre)
Dr. G.E. van den Bosch, paediatrician–neonatologist (Sophia Children’s Hospital, Erasmus University Medical Centre)
Prof. Dr. L.S. de Vries, paediatrician–neonatologist and paediatric neurologist (Willem-Alexander Children’s Hospital, Leiden University Medical Centre)*

**1.1 What is your professional role?**

1. (Paediatric) neurosurgeon
2. Paediatrician–neonatologist
3. Paediatric neurologist

**1.2 In which centre are you currently working?**

1. Amsterdam University Medical Centre – Emma Children’s Hospital
2. Erasmus University Medical Centre – Sophia Children’s Hospital
3. Isala Zwolle
4. Leiden University Medical Centre – Willem-Alexander Children’s Hospital
5. Maastricht UMC+
6. Máxima Medical Centre
7. Radboud University Medical Centre – Amalia Children’s Hospital
8. University Medical Centre Groningen – Beatrix Children’s Hospital
9. University Medical Centre Utrecht – Wilhelmina Children’s Hospital

### 1.3 How frequently is cranial ultrasonography routinely performed for preterm infants with GMH-IVH (grades 3 and 4) to monitor post-haemorrhagic ventricular dilatation (PHVD)?

1. Every 1–2 days
2. Every 3–4 days
3. Weekly
4. Biweekly
5. Other, namely: (open response)
6. Unknown

### 1.4 How is the diagnosis of PHVD established in your centre?

1. Based solely on clinical signs of increased intracranial pressure and/or increased head circumference
2. Based solely on radiological features on cranial ultrasonography (e.g., increased ventricular size, ballooning of the ventricles, or objective ventricular measurements)
3. Based on a combination of clinical and radiological features
4. Other, namely: (open response)

### 1.5 Which cranial ultrasonography features are used to establish the diagnosis of PHVD?

1. Only subjective assessment of increased ventricular size and/or ballooning of the lateral ventricles
2. Only objective measurement of ventricular size (ventricular index, anterior horn width, and/or thalamo-occipital distance)
3. Both subjective and objective assessment of ventricular size
4. Other, namely: (open response)
5. Unknown

### 1.6 Which ventricular measurements are routinely used for the diagnosis of PHVD?

1. Ventricular index > p97
2. Ventricular index > p97 + 4 mm
3. Anterior horn width > 6 mm
4. Anterior horn width > 10 mm
5. Thalamo-occipital distance > 25 mm
6. Ventricular index > p97 and anterior horn width > 6 mm
7. Ventricular index approaching p97 + 4 mm and anterior horn width > 10 mm
8. Ventricular index > p97 + 4 mm and anterior horn width > 10 mm
9. Ventricular index > p97, anterior horn width > 6 mm, and/or thalamo-occipital distance > 25 mm
10. Ventricular index approaching p97 + 4 mm, anterior horn width > 10 mm, and/or thalamo-occipital distance > 25 mm
11. Ventricular index > p97 + 4 mm, anterior horn width > 10 mm, and/or thalamo-occipital distance > 25 mm
12. Other, namely: (open response)
13. Unknown

### 1.7 On average, how many PHVD patients are treated in your centre per year (including patients without treatment indication, temporary, and permanent interventions) over the past three years?

1. 0–5
2. 6–10
3. 11–20
4. 20
5. Unclear/difficult to estimate

### 1.8 Which factors primarily determine the decision to initiate treatment for PHVD in your centre?

1. Only subjective assessment of increased ventricular size and/or ballooning of lateral ventricles
2. Only objective measurement of ventricular size (VI and AHW)
3. Both subjective and objective assessment of ventricular size
4. Other, namely: (open response)
5. Unknown

### 1.9 How frequently is cranial ultrasonography routinely performed in preterm infants with PHVD to determine the initiation of intervention(s)?

1. Every 1–2 days
2. Every 3–4 days
3. Weekly
4. Biweekly
5. Other, namely: (open response)
6. Unknown

### 1.10 If the initiation of therapy in your centre is determined by lateral ventricle measurements, which ventricular parameters inform this decision, and what threshold values are used?

1. Ventricular index > p97
2. Ventricular index approaching p97 + 4 mm
3. Ventricular index > p97 + 4 mm
4. Anterior horn width > 6 mm
5. Anterior horn width > 10 mm
6. Ventricular index > p97 and anterior horn width > 6 mm
7. Ventricular index approaching p97 + 4 mm and anterior horn width > 6 mm
8. Ventricular index > p97 + 4 mm and anterior horn width > 10 mm
9. Ventricular index > p97, anterior horn width >6 mm, and/or thalamo-occipital distance > 25 mm
10. Ventricular index approaching p97 + 4 mm, anterior horn width >10 mm, and/or thalamo-occipital distance >25 mm
11. Ventricular index > p97 + 4 mm, anterior horn width > 10 mm, and/or thalamo-occipital distance > 25 mm
12. Other, namely: (open response)
13. Unknown

**1.11 What volume of CSF (ml/kg) is generally aimed to be removed during a lumbar puncture?**

1. < 5 ml/kg
2. 5-10 ml/kg
3. > 10 ml/kg
4. Unknown

### 1.12 In which position are lumbar punctures performed in your centre?

1. Only supine
2. Only seated
3. Both positions
4. Unknown

### 1.13 What is the maximum number of lumbar punctures typically performed for PHVD, assuming CSF can be drained successfully and the patient tolerates the procedure, before proceeding to another (temporary or permanent) intervention?

1. 1
2. 2
3. 3
4. 4
5. 5
6. 6
7. 7 or more
8. Unknown

### 1.14 If a ventricular reservoir is used, which healthcare professional(s) perform(s) the punctures in your centre? (multiple options possible)

1. (Paediatric) neurosurgeon
2. (Paediatric) neurologist
3. Neonatologist
4. Residents
5. Physician assistant / nurse practitioner
6. Nurse
7. Other, namely: (open answer)
8. Unknown

### 1.15 How often is a ventricular access reservoir (VAR) punctured on average at the start of VAR punctures?

1. Less than 2×/week
2. 2–4×/week
3. 4×/week
4. Once per day
5. Twice per day
6. Other, namely: (open answer)
7. Unknown

### 1.16 How often are microbiological cultures obtained in your centre after VAR placement?

1. Once per week
2. Twice per week
3. Three times per week
4. More than three times per week
5. Never
6. Unknown

### 1.17 How frequently is cranial ultrasonography performed to determine the volume of CSF to be removed from a VAR?

1. Daily
2. Every other day
3. Twice per week
4. Less than twice per week

### 1.18 Is a MRI routinely performed in your centre prior to placement of a VAR or before performing a permanent CSF diversion procedure?

1. Yes, only before a temporary CSF diversion procedure
2. Yes, only before a permanent CSF diversion procedure
3. Yes, before both temporary and permanent CSF diversion procedures
4. No
5. Unknown

### 1.19 What is the maximum duration of VAR punctures before proceeding to permanent CSF diversion treatment (stoma/shunt), assuming weight and laboratory values (erythrocytes and protein levels) are within normal ranges?

1. 1–2 weeks
2. 3–4 weeks
3. 4 weeks
4. 4–6 weeks
5. 6 weeks
6. Longer than 6 weeks
7. Never longer than 6 weeks
8. Dependant on clinical condition or imaging, possibly longer than 6 weeks
9. Other, namely: (open answer)
10. Unknown

### 1.20 Is a “drain challenge” performed (e.g. temporarily stopping serial punctures) prior to a permanent CSF diversion procedure?

1. Yes
2. No
3. Unknown

**1.21 If possible, we kindly ask you to upload the PHVD treatment protocol of your centre to allow for comparison of potential local amendments.**

### 1.22 Are long-term neurodevelopmental outcomes systematically followed in PHVD patients? (Select all that apply)

1. Yes, systematically via neonatology
2. Yes, systematically via neurosurgery
3. Yes, systematically via paediatric neurology
4. Only in selected patients
5. No
6. Unknown

### 1.23 Which developmental assessment tools are used in your centre during the follow-up of children with PHVD? (multiple options possible)

1. Bayley Scales of Infant and Toddler Development (BSID)
2. Movement Assessment Battery for Children (M-ABC)
3. Wechsler Preschool and Primary Scale of Intelligence (WPPSI)
4. Other age-appropriate motor/cognitive assessments
5. Other, namely: (open response)
6. Unknown

### 1.24 Are you interested in participating in a national PHVD registry?

1. Yes
2. No
3. Maybe, under certain conditions, namely:

### 1.25 Which topics do you consider to have the highest priority for future PHVD research? (select a maximum of 3)

1. Optimal timing of the first intervention
2. Optimal timing of permanent CSF diversion
3. Role of third ventriculocisternostomy in neonates
4. Use of neuroendoscopic lavage
5. Prediction of neurological developmental outcomes
6. Benchmarking between centres (e.g., number of drains, drain revisions, infections, short- and long-term (neurological) outcomes)
7. Other, namely: (open answer)

### 1.26 Do you provide consent for your anonymized survey responses to be included in a potential scientific publication? If consent is provided, you will automatically be invited as a co-author.

### 1. Yes

2. No

**1.27 Are there specific challenges or innovative approaches related to PHVD that you would like to share?**

**1.28 Do you have any other suggestions, wishes, or comments for a PHVD consortium?**
