# Supplementary material for: Evaluation of nationwide variation in post-haemorrhagic ventricular dilatation management following national guideline implementation in the Netherlands
Source: Childs Nerv Syst. 2026 Sep 25;42(1):375. doi: 10.1007/s00381-026-07477-5 (PMC13615105; doi:10.1007/s00381-026-07477-5)
Supplement: Supplementary file 2 [file 381_2026_7477_MOESM2_ESM.docx]

**ONLINE RESOURCE 2. SURVEY PAEDIATRIC NEUROSURGEONS**

**JOURNAL**

Child’s Nervous System

**TITLE**

Evaluation of Nationwide Variation in Post-Haemorrhagic Ventricular Dilatation Management Following National Guideline Implementation in the Netherlands

**AUTHORS**

J.P.F. Bertens^1,2^, P.A. Woerdeman^3^, L.S. de Vries^4^, G.E. van den Bosch^5^, S.J. Steggerda^4^, J.K.H. Spoor^1^, L.S. Smit^5,6^, D.R. Buis^7^, R.W. Koot^8^, M.W. Aalbers^9^, H.R. Jeltema^10^, J. van Aalst^11^, N.E. van der Aa^12^, M.L. Tataranno^12^, L.C. Weeke^12,13^, H.J. ter Horst^14^, T.R. de Haan^15^, R.J. Vermeulen^16^, K.P. Dijkman^17^, S.M. Mulder-de Tollenaer^18^, E. Roze^5*^, O.H.J. Eelkman Rooda^1*^.

**These authors contributed equally.*

**AFFILIATIONS**

^1^Division of Paediatric Neurosurgery, Department of Neurosurgery, Sophia Children's Hospital, Erasmus University Medical Centre, Rotterdam, The Netherlands

^2^Department of Neurosurgery, Amsterdam University Medical Centre, Amsterdam, The Netherlands

^3^Division of Neuroscience, Department of Neurology and Neurosurgery, Paediatric Neurosurgery Wilhelmina Children's Hospital, University Medical Centre Utrecht, Utrecht, The Netherlands

^4^Division of Neonatology, Department of Paediatrics, Willem-Alexander Children's Hospital, Leiden University Medical Centre, Leiden, The Netherlands

^5^Division of Neonatology, Department of Neonatal and Paediatric Intensive Care, Sophia Children's Hospital, Erasmus University Medical Centre, Rotterdam, The Netherlands

^6^Division of Paediatric Neurology, Department of Neurology, Sophia Children's Hospital, Erasmus University Medical Centre, Rotterdam, The Netherlands

^7^Division of Paediatric Neurosurgery, Department of Neurosurgery, Emma Children's Hospital, Amsterdam University Medical Centre, Amsterdam, The Netherlands

^8^Division of Paediatric Neurosurgery, Department of Neurosurgery, Willem-Alexander Children's Hospital, Leiden University Medical Centre, Leiden, The Netherlands

^9^Division of Paediatric Neurosurgery, Department of Neurosurgery, Amalia Children's Hospital, Radboud University Medical Centre, Nijmegen, The Netherlands

^10^Division of Paediatric Neurosurgery, Department of Neurosurgery, Beatrix Children's Hospital, University Medical Centre Groningen, Groningen, The Netherlands

^11^Division of Paediatric Neurosurgery, Department of Neurosurgery, Mosakids Children's Hospital, Maastricht University Medical Centre, Maastricht, The Netherlands

^12^Department of Neonatology, Wilhelmina Children’s Hospital, University Medical Centre Utrecht, Utrecht, The Netherlands

^13^Division of Neonatology, Department of Paediatrics, Amalia Children's Hospital, Radboud University Medical Centre, Nijmegen, The Netherlands

^14^Department of Paediatrics, Beatrix Children's Hospital, University Medical Centre Groningen, Groningen, The Netherlands

^15^Division of Neonatology, Department of Paediatrics, Emma Children's Hospital, Amsterdam University Medical Centre, Amsterdam, The Netherlands

^16^Department of Paediatrics, Mosakids Children's Hospital, Maastricht University Medical Centre, Maastricht, The Netherlands

^17^Division of Neonatology, Department of Paediatrics, Máxima Medical Centre, Veldhoven, The Netherlands

^18^Division of Neonatology, Department of Paediatrics, Isala Women's and Children's Hospital, Zwolle, The Netherlands

**CORRESPONDING AUTHOR**

JPF Bertens^1,2^, [J.p.f.bertens@amsterdamumc.nl](mailto:J.p.f.bertens@amsterdamumc.nl)

**ENGLISH TRANSLATION OF THE ORIGINAL DUTCH SURVEY**

Dear colleague,

We kindly invite you to complete this survey (on behalf of your centre*) with the aim of gaining insight into the current treatment practices for post-haemorrhagic ventricular dilatation (PHVD) in neonates in Dutch (university) medical centres. Your input will help to map variation in practice, identify potential bottlenecks in PHVD care, and facilitate the development of joint research initiatives and future guidelines. The survey consists of 25 questions and takes approximately 10–15 minutes to complete. You will also be given the opportunity to indicate whether you would like to be involved in future research initiatives, if you wish.

**We request each centre to designate one paediatric neurosurgeon and one neonatologist/paediatric neurologist to complete the survey on behalf of the department.*

Kind regards,

*Dr. O.H.J. Eelkman Rooda, paediatric neurosurgeon (Erasmus University Medical Centre and Princess Máxima Centre)
Dr. E. Roze, paediatrician–neonatologist and paediatric neurologist (Sophia Children’s Hospital, Erasmus University Medical Centre)
Dr. S.J. Steggerda, paediatrician–neonatologist (Willem-Alexander Children’s Hospital, Leiden University Medical Centre)
Dr. G.E. van den Bosch, paediatrician–neonatologist (Sophia Children’s Hospital, Erasmus University Medical Centre)
Prof. Dr. L.S. de Vries, paediatrician–neonatologist and paediatric neurologist (Willem-Alexander Children’s Hospital, Leiden University Medical Centre)*

**1.1 What is your professional role?**

1. (Paediatric) neurosurgeon
2. Paediatrician–neonatologist
3. Paediatric neurologist

**1.2 In which centre are you currently working?**

1. Amsterdam University Medical Centre – Emma Children’s Hospital
2. Erasmus University Medical Centre – Sophia Children’s Hospital
3. Isala Zwolle
4. Leiden University Medical Centre – Willem-Alexander Children’s Hospital
5. Maastricht UMC+
6. Máxima Medical Centre
7. Radboud University Medical Centre – Amalia Children’s Hospital
8. University Medical Centre Groningen – Beatrix Children’s Hospital
9. University Medical Centre Utrecht – Wilhelmina Children’s Hospital

### 1.3 Which initial temporary interventions are routinely applied in your centre for PHVD? (multiple options possible)

1. Serial lumbar punctures
2. Serial ventricular/fontanel punctures
3. Placement of a ventricular access reservoir (VAR)
4. Placement of a subgaleal drain
5. Placement of an external ventricular drain (EVD)
6. Neuroendoscopic lavage
7. Other, namely: (open answer)
8. Unknown

### 1.4 What volume of CSF (ml/kg) is generally aimed to be removed during a lumbar puncture?

1. < 5 ml/kg
2. 5–10 ml/kg
3. 10 ml/kg
4. Unknown

### 1.5 Does your centre apply strict criteria for the placement of a VAR? (open question)

### 1.6 If lumbar puncture(s) have not improved or stabilized the PHVD, which of the following modalities is the preferred intervention in your centre prior to permanent shunting?

1. Serial ventricular/fontanel punctures
2. Placement of VAR
3. Placement of a subgaleal drain
4. Placement of EVD
5. Neuroendoscopic lavage
6. Other, namely: (open answer)
7. Unknown

### 1.7 How many PHVD patients in your centre undergo a first (temporary or permanent) CSF diversion procedure (this includes the entire spectrum, e.g., ventricular access device, ETV, subgaleal catheter, etc.) per year?

1. 0–5
2. 6–10
3. 11–20
4. 20
5. Unclear/difficult to estimate

### 1.8 What is the objective of each intervention for PHVD in your centre? (multiple options possible)

1. Relief of clinical symptoms of increased intracranial pressure and/or slowing the increase in head circumference to < 2 cm/week, if present
2. Prevention of further ventricular dilatation
3. Reduction of ventricular size
4. Reduction of ventricular size, specifically ventricular index < p97 + 4 mm
5. Reduction of ventricular size, specifically ventricular index < p97
6. Reduction of ventricular size, specifically anterior horn width < 10 mm
7. Reduction of ventricular size, specifically anterior horn width < 6 mm
8. Unknown

### 1.9 If a ventricular reservoir is used, which healthcare professional(s) perform(s) the punctures in your centre? (multiple options possible)

1. (Paediatric) neurosurgeon
2. (Paediatric) neurologist
3. Neonatologist
4. Residents
5. Physician assistant / nurse practitioner
6. Nurse
7. Other, namely: (open answer)
8. Unknown

### 1.10 How often is a ventricular access reservoir (VAR) punctured on average at the start of VAR punctures?

1. Less than 2×/week
2. 2–4×/week
3. 4×/week
4. Once per day
5. Twice per day
6. Other, namely: (open answer)
7. Unknown

### 1.11 How often are microbiological cultures obtained in your centre after VAR placement?

1. Once per week
2. Twice per week
3. Three times per week
4. More than three times per week
5. Never
6. Unknown

### 1.12 Is a MRI routinely performed in your centre prior to placement of a VAR or before performing a permanent CSF diversion procedure?

1. Yes, only before a temporary CSF diversion procedure
2. Yes, only before a permanent CSF diversion procedure
3. Yes, before both temporary and permanent CSF diversion procedures
4. No
5. Unknown

### 1.13 What is the maximum duration of VAR punctures before proceeding to permanent CSF diversion treatment (stoma/shunt), assuming weight and laboratory values (erythrocytes and protein levels) are within normal ranges?

1. 1–2 weeks
2. 3–4 weeks
3. 4 weeks
4. 4–6 weeks
5. 6 weeks
6. Longer than 6 weeks
7. Never longer than 6 weeks
8. Dependant on clinical condition or imaging, possibly longer than 6 weeks
9. Other, namely: (open answer)
10. Unknown

### 1.14 What is the preferred treatment for permanent CSF drainage for PHVD in your centre?

1. Ventriculoperitoneal shunt
2. Endoscopic third ventriculostomy (ETV)
3. ETV combined with choroid plexus coagulation (ETV + CPC)
4. Other combination strategy, namely: (open answer)
5. Lavage/suction/ultrasonic aspirator device (Cavitron Ultrasonic Surgical Aspirator, CUSA)
6. Unknown

### 1.15 What are the criteria for transitioning from a VAR to a VP shunt? (multiple options possible)

1. Weight
2. Protein level in CSF
3. Blood in CSF
4. Combination, namely: (open answer)
5. Other, namely: (open answer)
6. Unknown

### 1.16 Is ETV applied in neonates with PHVD?

1. Routinely if anatomically feasible
2. Only for specific indications (obstructive component, e.g., aqueductal stenosis)
3. Sometimes, rarely, or never
4. Not applicable

### 1.17 What is the minimum weight at which you consider ETV in PHVD?

1. > 2 kg
2. > 2.5 kg
3. > 3 kg
4. > 3.5 kg
5. Unknown

### 1.18 What is the minimum weight at which you consider a VP shunt in PHVD?

1. > 2 kg
2. > 2.5 kg
3. > 3 kg
4. > 3.5 kg
5. Unknown

**1.19 If possible, we kindly ask you to upload the PHVD treatment protocol of your centre to allow for comparison of potential local amendments.**

### 1.20 Are complications (e.g. infections, haemorrhage, and drain dysfunction) of PHVD interventions systematically recorded in your centre?

1. Yes, via neurosurgical department in the electronic patient record
2. Yes, via neonatology department in the electronic patient record
3. Yes, in the electronic patient record
4. Yes, in a separate digital registry
5. No
6. Unknown

### 1.21 Are you interested in participating in a national PHVD registry?

1. Yes
2. No
3. Maybe, under certain conditions, namely:

### 1.22 Which topics do you consider to have the highest priority for future PHVD research? (select a maximum of 3)

1. Optimal timing of the first intervention
2. Optimal timing of permanent CSF diversion
3. Role of third ventriculocisternostomy in neonates
4. Use of neuroendoscopic lavage
5. Prediction of neurological developmental outcomes
6. Benchmarking between centres (e.g., number of drains, drain revisions, infections, short- and long-term (neurological) outcomes)
7. Other, namely: (open answer)

### 1.23 Do you provide consent for your anonymized survey responses to be included in a potential scientific publication? If consent is provided, you will automatically be invited as a co-author.

### 1. Yes

2. No

**1.24** **Are there specific challenges or innovative approaches related to PHVD that you would like to share?**

**1.25** **Do you have any other suggestions, wishes, or comments for a PHVD consortium?**
